# Autoimmune conditions and hairy cell leukemia: an exploratory case-control study

**DOI:** 10.1186/1756-8722-3-35

**Published:** 2010-10-04

**Authors:** Lesley A Anderson, Eric A Engels

**Affiliations:** 1 Cancer Epidemiology and Health Services Research Group, Centre for Public Health, School of Medicine, Dentistry and Biomedical Sciences, Queen's University Belfast, Northern Ireland, UK; 2 Infection and Immunoepidemiology Branch, Division of Cancer Epidemiology and Genetics, National Cancer Institute, Rockville, MD, USA

## Abstract

**Background:**

Case reports suggest that hairy cell leukemia (HCL) may be associated with autoimmune conditions, however no systematic investigations in this area have been undertaken.

**Methods:**

Using the United States Surveillance, Epidemiology, and End Results Medicare linked database, we conducted an exploratory study comparing autoimmune conditions in 418 HCL cases (aged ≥65 years) and 160,086 controls.

**Results:**

Overall, the proportion with autoimmune conditions was similar between HCL cases and controls (n = 79 (18.9%) and n = 29,284 (18.3%), respectively). Before diagnosis/selection, there was no overall difference in the prevalence of autoimmune conditions in HCL cases (n = 37, 8.9%) compared with controls (n = 14,085, 8.8%), p = 0.969. However, compared with controls, HCL cases more frequently had sarcoidosis (OR 9.6, 95%CI 2.4-39.5), Sjögren syndrome (OR 6.1, 95%CI 2.0-19.3) and erythema nodosum (OR 37, 95%CI 4.9-284) before diagnosis. Autoimmune conditions were also more common in HCL cases than controls around the time of diagnosis/selection (p < 0.001) but not subsequently.

**Conclusions:**

The findings do not support an overall relationship between autoimmune conditions and HCL, although the association with some autoimmune conditions prior to HCL diagnosis may warrant further investigation. Our findings also suggest that autoimmune conditions in HCL patients may be detected around the time of diagnosis.

## Introduction

Hairy cell leukemia (HCL) is a rare, indolent, B-cell neoplasm, accounting for approximately 2% of all non-Hodgkin lymphomas (NHLs) in the U.S. [1]. Organic solvents and some medical conditions including anemia could be related to development of HCL [2]. Factors affecting the immune system, including autoimmune conditions, are associated with an elevated risk of several other NHL subtypes [3]. Case reports have described the occurrence of autoimmune conditions antecedent to and following diagnosis or treatment of HCL [4-8], suggesting that autoimmune conditions may be associated with this malignancy.

Nonetheless, no prior study has systematically assessed associations between a range of autoimmune conditions and HCL. We therefore conducted an exploratory study using linked U.S. data from the Surveillance, Epidemiology, and End Results (SEER) cancer registry program and Medicare to investigate the relation between a range of autoimmune conditions and HCL, separately examining the periods before, at, and after HCL diagnosis.

## Methods

The SEER-Medicare Assessment of Hematopoietic Malignancy Risk Traits (SMAHRT) Study is a population-based study of hematopoietic malignancies in elderly adults, using SEER-Medicare linked data [9]. The SEER program (1973-2002) includes data on cancer cases covering approximately 25% of the U.S. population for the most recent years. Medicare provides federally funded health insurance for approximately 97% of persons aged ≥65 years in the U.S. All Medicare beneficiaries have Part A coverage for hospital inpatient care, and approximately 96% subscribe to Part B coverage for physician and outpatient services. The SEER-Medicare database linkage includes Medicare enrollment and claims data (1986-2002) for SEER cancer cases diagnosed through December 2002, and for a 5% random sample of Medicare beneficiaries residing in SEER registry areas [10].

The SMAHRT Study includes as cases individuals from the SEER-Medicare database with a hematopoietic malignancy. Cases were aged 67-99 years at diagnosis, with at least 12 months of prior Part A and Part B coverage and without enrollment in a health maintenance organization to ensure adequate accrual of information on medical conditions prior to malignancy diagnosis. For the present analyses, we included cases with HCL (International Classification of Diseases for Oncology Version 3, code 9940) diagnosed between 1987 and 2002.

The SMAHRT Study includes two controls per hematopoietic malignancy case (n = 83,113), selected from the 5% random sample of Medicare beneficiaries. Controls were frequency matched to all hematopoietic malignancy cases by calendar year of diagnosis, age in five categories (67-69, 70-74, 75-79, 80-84, 85-99 years) and gender. Cases and controls with a prior diagnosis of human immunodeficiency virus infection were excluded. All SMAHRT Study controls were utilized in the present analysis (n = 160,086).

Using Medicare data, we searched for claims for specific autoimmune conditions. Subjects were classified as having an autoimmune condition if they had ≥1 hospital claim, or ≥2 physician or outpatient claims for the condition at least 30 days apart. Autoimmune conditions with a prevalence of ≥0.1% in control subjects were included in the study. Autoimmune conditions were categorized as being before, at, or after "diagnosis/selection" according to whether the above definition was first met in the period >12 months before, from 12 months before until 12 months after, or >12 months after HCL diagnosis (in cases) or selection (in controls).

Chi-square tests were used to compare the overall proportion of cases and controls with autoimmune conditions. Logistic regression was used to calculate ORs comparing the prevalence of autoimmune conditions before diagnosis/selection in cases and controls. Analyses were adjusted for the matching factors and race (white, non-white). Additional analyses were restricted to whites only or to females only. Proportional hazards regression, adjusting for matching factors, was used to compare the time to diagnosis of autoimmune conditions after HCL diagnosis/control selection with patients censored at the date of death or end of study date (December 31, 2002), whichever occurred earlier.

## Results

The study included 418 HCL cases (Table 1). Compared with controls (n = 160,086), HCL cases were more likely to be male, younger, and white. Duration of Medicare coverage and the number of prior physician and outpatient claims were similar for cases and controls. Controls tended to have more hospital claims than HCL cases.

**Table 1:** Characteristics of hairy cell leukemia cases and controls.

	Hairy cell leukemia cases (n = 418)	Controls (n = 160,086)	P-value
Gender			< 0.001
Male	285 (68.2%)	78,620 (49.1%)	
Female	133 (31.8%)	81,466 (50.9%)	
Age, years			0.003
67-69	70 (16.8%)	19,135 (12.0%)	
70-74	122 (29.2%)	40,611 (25.4%)	
75-79	96 (23.0%)	41,724 (26.1%)	
80-84	76 (18.2%)	32,091 (20.1%)	
85-99	54 (12.9%)	26,902 (16.6%)	
Selection year			0.401
1987-1996	203 (48.6%)	71,396 (44.6%)	
1997-1999	69 (16.5%)	26,946 (16.8%)	
2000-2001	97 (23.2%)	40,750 (25.5%)	
2002	49 (11.7%)	20,994 (13.1%)	
Race/ethnicity			< 0.001
White	390 (93.3%)	135,280 (84.5%)	
Black	10 (2.4%)	10,897 (6.8%)	
Asian	5 (1.2%)	5,629 (3.5%)	
Hispanic	< 5	3,408 (2.1%)	
Native American Indian	< 5	448 (0.2%)	
Other/unknown	8 (1.9%)	4,424 (2.8%)	
Duration of Medicare benefits	177 (42.3%)	62,264 (38.9%)	0.359
12-57 months	99 (23.7%)	36,842 (23.0%)	
58-93 months	71 (17.0%)	30,696 (19.2%)	
94-136 months	71 (17.0%)	30,284 (18.9%)	
≥137 months			
Number of physician claims*			0.590
0-20	183 (43.8%)	68,324 (42.7%)	
21-57	80 (19.1%)	30,532 (19.1%)	
58-127	86 (20.6%)	30,763 (19.2%)	
≥128	69 (16.5%)	30,467 (19.0%)	
Number of outpatient claims*			0.808
0	166 (39.7%)	62,453 (39.0%)	
1-3	81 (19.4%)	32,154 (20.1%)	
4-7	61 (14.6%)	21,293 (13.3%)	
8-15	56 (13.4%)	20,722 (12.9%)	
≥16	54 (12.9%)	23,464 (14.7%)	
Number of hospital claims*			0.035
0	235 (56.2%)	87,059 (54.4%)	
1	90 (21.5%)	28,505 (17.8%)	
2-3	49 (11.7%)	25,255 (15.7%)	
≥4	44 (10.5%)	19,267 (12.0%)	

Notes:

All entries are number of subjects (%). When the number of subjects was less than 5, the entry indicates " < 5" rather than specifying the number, in accordance with the SEER-Medicare data use agreement.

*The number of claims excludes the one year period prior to diagnosis/selection.

Overall, 79 (18.9%) HCL cases and 29,284 (18.3%) controls had at least one of the autoimmune conditions presented in Table 2, p = 0.749. During the period before diagnosis/selection, there was no overall difference in the prevalence of autoimmune conditions in HCL cases (n = 37, 8.9%) compared with controls (n = 14,085, 8.8%), p = 0.969. Nonetheless, among specific autoimmune conditions, sarcoidosis (OR 9.6, p = 0.002), Sjögren syndrome (OR 6.1, p = 0.002), and erythema nodosum (OR 37, p < 0.001) were more common in cases than controls, despite small numbers (Table 2). Most cases were diagnosed with the respective autoimmune condition in the 1-5 year period before diagnosis. When we restricted our analyses to whites only or females only the point estimates were similar (data not shown).

**Table 2:** Risk of autoimmune conditions in hairy cell leukemia cases compared to controls

Autoimmune condition	Controls with autoimmune condition	HCL cases with autoimmune condition	Association before HCL dx/control selection	Association at HCL dx/control selection	Association after HCL dx/control selection
	No. (%)	No. (%)	Odds Ratio (95%CI)*	Odds Ratio (95%CI)*	Hazard Ratio (95%CI)*
Addison disease	614 (0.4)	< 5	2.1 (0.3-15.2)	-	1.1 (0.9-1.3)
Alopecia areata	169 (0.1)	0	-	-	-
Ankylosing spondylitis	251 (0.2)	< 5	-	12.2 (1.7-90.7)	-
Chronic rheumatic heart disease	10,405 (6.5)	23 (5.5)	0.8 (0.4-1.7)	1.4 (0.6-3.2)	1.0 (1.0-1.1)
Crohn disease	546 (0.3)	< 5	-	10.2 (2.5-41.7)	-
Discoid lupus erythematosus	273 (0.2)	0	-	-	-
Erythema nodosum	86 (0.1)	< 5	37 (4.9-284)	-	-
Giant cell arteritis	899 (0.6)	< 5	2.2 (0.6-9.1)	-	-
Goodpasture syndrome	13 (0.0)	0	-	-	-
Graves disease	727 (0.5)	0	-	-	-
Hashimoto thyroditis	588 (0.4)	< 5	1.7 (0.2-12.1)	10.3 (2.5-42.3)	-
Systemic lupus erythermatosus	545 (0.3)	< 5	1.8 (0.3-13.0)	7.2 (2.3-22.8)	1.1 (0.9-1.4)
Meniere syndrome	800 (0.5)	< 5	1.0 (0.1-7.2)	-	1.1 (0.9-1.4)
Myasthenia gravis	232 (0.1)	0	-	-	-
Pernicious anemia	5,229 (3.3)	17 (4.1)	1.1 (0.5-2.8)	3.4 (1.6-7.1)	1.0 (0.9-1.1)
Polymyositis	206 (0.1)	0	-	-	-
Polymyalgia rheumatica	2,721 (1.7)	9 (2.6)	1.5 (0.5-3.9)	1.1 (0.2-8.0)	1.1 (1.0-1.2)
Psoriasis	2,655 (1.7)	< 5	0.8 (0.2-2.3)	1.0 (0.1-7.0)	-
Rheumatoid arthritis	6,557 (4.2)	12 (4.1)	1.3 (0.7-2.5)	1.0 (0.2-3.9)	0.9 (0.7-1.1)
Sarcoidosis	167 (0.1)	< 5	9.6 (2.4-39.5)		-
Localised scleroderma	345 (0.2)	0	-	-	-
Systemic sclerosis	156 (0.1)	0	-	-	-
Sjögren syndrome	472 (0.3)	5 (1.2)	6.1 (2.0-19.3)	7.9 (1.1-57.5)	1.2 (0.9-1.5)
Ulcerative colitis	951 (0.6)	< 5	0.8 (0.1-5.6)	2.8 (0.4-19.8)	-

Notes:

Abbreviations: HCL hairy cell leukemia, dx diagnosis

The table includes only autoimmune conditions that had a prevalence of greater than 0.1% in controls. Associations that were significant at p < 0.05 are underlined. Observations where the number of exposed cases or controls is between 1 and 4 are listed as " < 5" to preserve subjects' anonymity, in accordance with the SEER-Medicare data use agreement.

Associations that are significant at p < 0.05 are underlined.

* Analyses were adjusted for age (67-69, 70-74, 75-79, 80-84 and 85-99 years), gender, race (white, non-white) and selection year (1987-1996, 1997-1999, 2000-2001, 2002).

In the period after diagnosis/selection, no autoimmune conditions occurred more frequently in HCL cases compared to controls, Table 2. Overall, 23 (5.5%) HCL cases and 11,993 (7.5%) controls had at least one autoimmune condition in this period, p = 0.122.

Autoimmune conditions were more common in HCL patients (n = 26, 6.2%) at the time of diagnosis than controls (n = 5,146, 3.2%), p < 0.001. Of the autoimmune conditions, ankylosing spondylitis, Crohn disease, Hashimoto thyroditis, systemic lupus erythematosus, pernicious anemia, and Sjögren syndrome were more commonly reported in cases than controls during this time period, Table 2.

## Discussion

This exploratory case-control study of 418 HCL cases is the first to examine associations with a diverse range of autoimmune conditions. The rarity of this NHL subtype presents a challenge for systematic epidemiologic study, and prior reports have mostly consisted of small case series or case reports. Although our study included more HCL cases than previous studies, most autoimmune conditions are also uncommon. Thus, the associations reported were based on few cases, which led to imprecise estimates.

Although we found no overall difference in the proportion of cases and controls with an autoimmune condition, there was some evidence for an excess of sarcoidosis, erythema nodosum and Sjögren syndrome occurring antecedent to HCL diagnosis. Overlap between these three conditions has previously been reported [11] and erythema nodosum [8,12] and sarcoidosis [8] have been reported in HCL patients. Sjögren syndrome and several other autoimmune conditions are associated with an increased risk of developing other NHL subtypes [3], which might be explained by antigen-driven chronic inflammation [13]. In addition, non-steroidal anti-inflammatory drugs have been associated with a three-fold increased risk of HCL [2]. While use of immunosuppressive medications to treat some autoimmune conditions has been linked to lymphoma, a recent study has suggested that the severity of the condition, not its treatment, is responsible for the added risk [14]. If certain autoimmune conditions do increase the risk of HCL, it may therefore be due to the autoimmune disease itself rather than its treatment. Most cases with these autoimmune conditions were diagnosed in the 1-5 year period prior to diagnosis suggesting the possibility that some of these conditions may be part of the early disease process of HCL. However, there were too few cases for us to reliably evaluate the time from first claim for the autoimmune condition until HCL diagnosis, which would have shed additional light.

In contrast to these restricted associations, we found a much broader range of associations between HCL and various autoimmune conditions at the time of diagnosis. This observation could have several explanations: causality in either direction (i.e., the autoimmune condition or its treatment leading to HCL, or HCL or its treatment leading to the autoimmune condition), the presence of a common underlying risk factor for both the autoimmune condition and HCL, or over diagnosis in HCL patients undergoing medical evaluation for HCL-related symptoms which would result in detection bias. Arguing against this last possibility, however, we note that the prevalence of non-immune-related medical conditions (including hypertension, hyperlipidemia, peptic ulcer disease, migraine, depression, and gastroesophageal reflux) were not significantly higher in HCL patients compared to controls at the time of diagnosis/selection (data not shown).

Limitations and strengths of our study should be considered. We relied upon Medicare claims to determine the presence of autoimmune conditions. To reduce the possibility of misdiagnosis, we only considered subjects as having an autoimmune condition if they had a hospital diagnosis, or at least two physician or outpatient claims at least 30 days apart. In addition, our study was limited to HCL cases aged >65 years, which comprise only 37.8% of U.S. cases [15]. Our results may not be generalizable to younger HCL cases. Furthermore, as noted above, the associations with sarcoidosis, Sjögren syndrome, and erythema nodosum although strong (ORs 9.6, 6.1 and 37, respectively) were based on few affected HCL cases and may have occurred by chance. Given the rarity of HCL, our study is the largest to date. Our study has several additional strengths, including population-based sampling of HCL cases, a large number of controls representative of the Medicare population, and availability of Medicare claims files, enabling the systematic evaluation of numerous autoimmune conditions.

## Conclusions

In conclusion, our study found little difference in the occurrence of autoimmune conditions between HCL cases and controls except at the time around HCL diagnosis. Despite small numbers of affected individuals, sarcoidosis, Sjögren syndrome and erythema nodosum were associated with an increased risk of subsequent HCL. Chance, chronic immune stimulation or medication used in the treatment of these autoimmune conditions may explain the findings. Further investigation of these exploratory findings may be warranted.

## Competing interests

The authors declare that they have no competing interests.

## Authors' contributions

EAE and LAA conceived the study idea, LAA conducted the statistical analyses, EAE and LAA wrote the manuscript. All authors read and approved the final manuscript.
